# Characterisation of a Novel Cell Line (ICR-SS-1) Established from a Patient-Derived Xenograft of Synovial Sarcoma

**DOI:** 10.3390/cells11152418

**Published:** 2022-08-04

**Authors:** William G. J. Kerrison, Jian Ning, Lukas Krasny, Amani Arthur, Nafia Guljar, Mark L. Elms, Amanda Swain, Robin L. Jones, Khin Thway, Paul H. Huang

**Affiliations:** 1 Division of Molecular Pathology, The Institute of Cancer Research, Sutton SM2 5NG, UK; 2 Division of Cancer Biology, The Institute of Cancer Research, London SW3 6JB, UK; 3 Sarcoma Unit, The Royal Marsden NHS Foundation Trust, London SW3 6JJ, UK; 4 Division of Clinical Studies, The Institute of Cancer Research, London SW3 6JB, UK

**Keywords:** synovial sarcoma, patient-derived xenograft, cancer therapeutics, soft tissue sarcoma, doxorubicin

## Abstract

Synovial sarcoma is a rare translocation-driven cancer with poor survival outcomes, particularly in the advanced setting. Previous synovial sarcoma preclinical studies have relied on a small panel of cell lines which suffer from the limitation of genomic and phenotypic drift as a result of being grown in culture for decades. Patient-derived xenografts (PDX) are a valuable tool for preclinical research as they retain many histopathological features of their originating human tumour; however, this approach is expensive, slow, and resource intensive, which hinders their utility in large-scale functional genomic and drug screens. To address some of these limitations, in this study, we have established and characterised a novel synovial sarcoma cell line, ICR-SS-1, which is derived from a PDX model and is amenable to high-throughput drug screens. We show that ICR-SS-1 grows readily in culture, retains the pathognomonic *SS18::SSX1* fusion gene, and recapitulates the molecular features of human synovial sarcoma tumours as shown by proteomic profiling. Comparative analysis of drug response profiles with two other established synovial sarcoma cell lines (SYO-1 and HS-SY-II) finds that ICR-SS-1 harbours intrinsic resistance to doxorubicin and is sensitive to targeted inhibition of several oncogenic pathways including the PI3K-mTOR pathway. Collectively, our studies show that the ICR-SS-1 cell line model may be a valuable preclinical tool for studying the biology of anthracycline-resistant synovial sarcoma and identifying new salvage therapies following failure of doxorubicin.

## 1. Introduction

Synovial sarcoma is a rare mesenchymal tumour type that accounts for 8–10% of all soft tissue sarcomas [1]. As with many other sarcoma subtypes, synovial sarcoma can occur in any anatomical site, but is most commonly found in the extremities [2]. It typically arises in adolescents and young adults although can affect patients of any age [3,4]. Synovial sarcoma is characterised by a pathognomonic translocation between chromosome X and 18 (X:18), which results in the expression of fusion proteins including SS18-SSX1, SS18-SSX2, and SS18-SSX4 [5,6,7]. Extensive work has shown that these fusion proteins play important roles in driving sarcomagenesis, such as regulating the biology of the SWI/SNF chromatin remodelling complex [8,9,10]. Patients with synovial sarcoma have poor outcomes with a 5-year survival of 50–60% [4,11,12] and have limited treatment options in the advanced/metastatic setting, including anthracyclines, ifosfamide, trabectedin, and pazopanib [13].

In order to identify new therapeutic strategies and gain a better understanding of the biology of this disease, it is necessary to develop preclinical models of synovial sarcoma. Given its rarity, there are very few synovial sarcoma cell line models available in public repositories, with only five identified in the most recent census of sarcoma cell line models [14]. Established cancer cell lines have been cultured on plastic for decades and due to genomic and phenotypic drift during serial passaging have diverged from the tumours for which they were derived [15]. This has contributed to poor reproducibility in preclinical findings which is likely to have played a role in the high failure rate of translating therapeutic discoveries into oncology clinical trials and regulatory approvals [16,17].

To bridge this gap, patient-derived xenografts (PDX), where tumours obtained from patients are serially passaged in mice, have emerged as a valuable tool for preclinical research as they retain much of the molecular and histopathological features of the original human tumour [18,19]. Several sarcoma PDX collections have been described encompassing multiple subtypes including synovial sarcoma [20,21,22]. However, PDX models have certain limitations including high costs required for animal maintenance, slow tumour growth, and variable engraftment rate, which collectively diminish the feasibility of undertaking large-scale genetic and drug screens. These limitations can be overcome by the establishment of matched PDX-derived cell lines which retain many of the genomic features of the PDX tumours, are easier to grow and manipulate in culture, and are amenable to high-throughput screens [18]. The development of matched PDX-derived cell lines is non-trivial due to the relatively high failure rate associated with establishing cell lines from tumour specimens [23,24] and there are only a few reported studies demonstrating success using this approach, including in breast, colorectal, and pancreatic cancers [21,25,26,27]. In sarcomas, matched PDX-derived lines have been established in osteosarcoma, Ewing’s sarcoma, clear cell sarcoma, and *CIC::DUX4* sarcoma [23,28,29,30].

In this study, we established a novel synovial sarcoma cell line, ICR-SS-1, which was derived from a PDX model from The Jackson Laboratory biorepository. We have further undertaken a comparative analysis of the drug response profiles of ICR-SS-1 versus two established commercially available synovial sarcoma cell lines (SYO-1 and HS-SY-II). To our knowledge, this is the first study to report a matched PDX-derived cell line for synovial sarcoma.

## 2. Materials and Methods

### 2.1. Patient-Derived Xenograft Model

**Figure 1:**
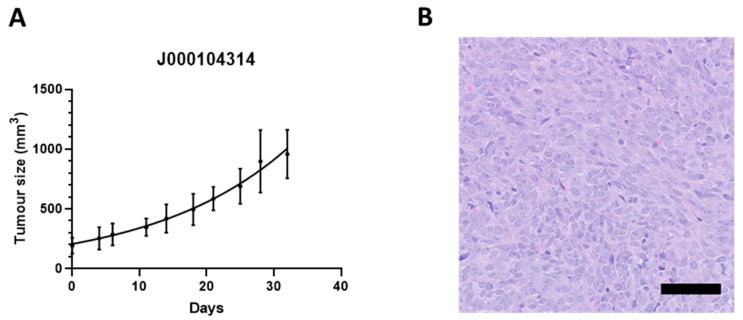
(**A**) Tumour growth curve of the J000104314 patient-derived xenograft model. *n* = 5. (**B**) H&E stain of tumour section from the J000104314 patient-derived xenograft model. Scale bar = 50 µm.

ICR-SS-1 was established from a publicly available PDX model (J000104314) deposited in The Jackson Laboratory biorepository (The Jackson Laboratory, http://tumor.informatics.jax.org/mtbwi/pdxDetails.do?modelID=J000104314, accessed on 17 June 2022). This PDX was derived from a tumour obtained from a 21-year-old male diagnosed with a grade 3 metastatic synovial sarcoma. This PDX model has been shown by The Jackson Laboratory to harbour the *SS18::SSX1* fusion gene. PDX tumours were serially passaged in NOD scid gamma (NSG) mice and tumour volume calculated by ½ × length × width^2^ (Figure 1A). Histology showed a hypercellular neoplasm composed of sheets of uniform spindle and ovoid cells, with nuclear overlapping, scanty cytoplasm, and minimal surrounding stroma. There was no discernible pleomorphism. This is in keeping with monophasic synovial sarcoma (Figure 1B). These tumours maintain the same histological features as the histology images deposited in The Jackson Laboratory biorepository database.

### 2.2. PDX Dissociation

To generate a cell suspension from the xenograft tumour, the tissue was minced and digested for 2 h at 37 °C in DMEM/Ham’s F12 1:1 with 15 mM HEPES, 0.1× insulin-transferrin selenium A (Gibco, Thermo Fisher Scientific, Waltham, MA, USA), 1× penicillin/streptomycin, 10 ng/mL EGF (Peprotech, London, UK), 10 µg/mL hydrocortisone (Sigma Aldrich, St. Louis, MO, USA), 0.5 mg/mL collagenase (Sigma Aldrich), 0.1 mg/mL hyaluronidase (Sigma Aldrich), 100 units/mL DNase I (Sigma Aldrich), 10 µM Y-27632 (LC Laboratories, Woburn, MA, USA), and 5% FBS (Gibco). Red blood cells were lysed using RBC lysis buffer (Invitrogen, Waltham, MA, USA) and remaining cells were incubated with 0.05% trypsin-EDTA (Gibco) at 37 °C. After trypsinisation, cells were treated with 1 mg/mL DNase I (Sigma Aldrich) at 37 °C, before passing through a 70 µm strainer. Mouse cell depletion beads (Miltenyi Biotec, Surrey, UK) were used to remove contaminating murine cells.

### 2.3. Cell Culture

Dissociated and mouse cell depleted PDX tumour cells were cultured in DMEM/Ham’s F12 1:1 with 15 mM HEPES, 1× penicillin/streptomycin, 2.4 mM L-glutamine, 5 µM Y-27632 (LC Laboratories), 5 µg/mL insulin (Sigma Aldrich), 400 ng/mL hydrocortisone (Sigma Aldrich), 10 ng/mL EGF (Peprotech), 250 ng/mL amphotericin B (Thermo Fisher Scientific), 9.62 ng/mL cholera toxin (Sigma Aldrich), and 10% FBS (Gibco). Following successful continuous growth for >10 passages in culture, the cell line was designated ICR-SS-1. HS-SY-II (from RIKEN BioResource Centre, Kyoto, Japan), SYO-1 (obtained from Dr Chris Lord, Institute of Cancer Research, London, UK), and NIH-3T3 (obtained from Dr Matilda Katan, University College London, London, UK) cells were cultured in DMEM supplemented with 1× penicillin/streptomycin and 10% FBS (Gibco). SK-UT-1 cells (obtained from Dr Priya Chudasama, German Cancer Research Centre, Heidelberg, Germany) were cultured in MEM supplemented with 1× penicillin/streptomycin and 10% FBS (Gibco). Cells were grown at 37 °C with 5% CO_2_. Medium was replenished twice weekly.

For spheroid formation, ICR-SS-1 cells (1000/well) were seeded into 96-well, round-bottom, ultra-low attachment plates (Corning). Plates were spun at 1000× *g* and grown at 37 °C with 5% CO_2_ for 3 days before imaging.

### 2.4. Cell Proliferation

ICR-SS-1 cells (1000/well) were seeded into 96-well, flat-bottom, black-walled plates (Greiner Bio-One, Frickenhausen, Germany). Plates were fixed in 10% neutral-buffered formalin solution (Sigma Aldrich) at respective timepoints and stained with 5 µg/mL Hoechst 33342 (Tocris, Tocris Bioscience, Bristol, UK). Plates were scanned and cells were counted using a Celigo imaging cytometer. Cell counts were normalised to day 1 and an exponential growth equation was fitted to the data using GraphPad Prism (GraphPad, v8.2.1) in order to determine doubling time.

### 2.5. SS18::SSX Fusion PCR

RNA was extracted from J000104314 tissue and ICR-SS-1 cells using RNeasy mini and QIAshredder kits (Qiagen, Germantown, MD, USA), following the manufacturer’s instructions, and used to generate cDNA via a Superscript III kit (Invitrogen). The HS-SY-II cell line was used as a positive control. PCR mixtures were made containing the common *SS18* forward primer and one of *SSX1*, *SSX2*, or *SSX4* reverse primers. *ACTB* was used as a loading control. Primer sequences are provided in Table 1 below.

Thermocycler conditions used for *SS18::SSX* or *ACTB* amplification are presented in Table 2 and Table 3 below. PCR products were run on 2% agarose gels with SYBR safe DNA gel stain (Invitrogel, Invitrogen) or ethidium bromide (Sigma Aldrich) and visualised using UV illumination.

### 2.6. Human and Mouse PTGER2 PCR

DNA was extracted from ICR-SS-1, NIH-3T3, and SK-UT-1 cells using a DNeasy blood and tissue kit (Qiagen), following the manufacturer’s instructions. NIH-3T3 was used as a mouse positive control and SK-UT-1 was used as a human positive control. PCR mixtures were made containing either a human- or mouse-specific *PTGER2* forward primer and a common reverse *PTGER2* primer. Primer sequences are provided in Table 4 below.

Thermocycler conditions used for human or mouse *PTGER2* amplification are presented in Table 5 below. PCR products were run on 1.5% agarose gels with SYBR safe DNA gel stain (Invitrogel) and visualised using UV illumination.

### 2.7. Short Tandem Repeat (STR) Analysis

Genomic DNA was harvested from J000104314 PDX tissue and ICR-SS-1 cells using a DNeasy blood and tissue kit (Qiagen), following the manufacturer’s instructions. DNA was quantified using a Qubit dsDNA HS assay kit, and STR profiles were then analysed via Eurofins cell line authentication service.

### 2.8. Proteomic Analysis

ICR-SS-1, HS-SY-II, and SYO-1 cells were seeded in T25 culture flasks and incubated for 72 h in order to reach 80% confluence. Cells were then lysed in 8 M urea and 0.1 M ammonium bicarbonate (ABC), and protein concentration was measured by bicinchoninic acid (BCA) assay. For each cell line, 40 ug of total protein was reduced with 10 mM dithiothreitol at 56 °C for 40 min and alkylated with 55 mM iodoacetamide at 25 °C for 30 min in the dark. After dilution to final concentration of 2 M urea and 0.1 M ABC, each sample was digested with 0.4 g of trypsin (Thermo Scientific) at 37 °C overnight. The resulting digest was acidified to pH < 4 by trifluoroacetic acid (TFA), desalted on Pierce C18 Spin Columns (Thermo Scientific) according to the manufacturer’s protocol, and dried in a SpeedVac.

Dried samples were resuspended in mobile phase A (2% acetonitrile, 0.1% formic acid), spiked with iRT peptides (Biognosys AG, Schlieren, Switzerland), and 2 ug of total peptides were loaded onto a 2 cm × 0.1 mm trap column self-packed with ReproSil Pur C18AQ (120 Å, 10 µm) beads. Sequential window acquisition of all theoretical mass spectra (SWATH)-mass spectrometry data were acquired using the same instrument parameters as previously described in Milighetti et al. [31]. The acquired data were integrated with a previously published dataset of synovial sarcoma, leiomyosarcoma, undifferentiated pleomorphic sarcoma, and dedifferentiated liposarcoma FFPE tissue samples from Milighetti et al. [31], and all data were analysed by DIA-NN (v1.8) software (accessed on 17 June 2022) [32] using a publicly available pan-human library [33]. The default settings with “match between runs” and “unrelated runs” was used for data processing. Oxidation of methionine, carbamidomethylation of cysteines, and N-term methionine excision were included as possible amino acid modifications with a maximum number of 5 modifications per peptide sequence. Quantified protein data were log2 transformed and quantile normalised in R using the proBatch package [34] and further processed in Perseus [35]. Proteins with >75% values in at least one sarcoma subtype or across all cell lines were retained. Missing values were imputed using the “replace missing values from normal distribution” tool in Perseus using default settings. The imputed dataset was then median-centred across all samples and visualised using two-way unsupervised clustering based on Pearson’s correlation coefficient.

Proteomic profiles of the three synovial sarcoma cell lines and FFPE tissue samples were analysed by significance analysis of microarrays (SAM) using the samR package [36] in R. For this, the log2 transformed and quantile normalised dataset was used. Data for cell lines and tissue samples were selected, technical replicates averaged, and proteins with more than 30% of missing values were removed. The resulting dataset was processed by samR using a delta score threshold of 0.77 to reach 1% FDR. Lists of positively and negatively regulated proteins were then separately subjected to over-representation analysis using the online tool g:Profiler [37] with the g:GOSt module for functional profiling (g:Profiler, https://biit.cs.ut.ee/gprofiler/gost, accessed on 17 June 2022) and the following setup: full list of identified proteins used as a background; Benjamini–Hochberg FDR method with 0.1 FDR threshold; GOBP and Hallmark gene set databases downloaded from MSigDB (Molecular Signatures Database, v7.5.1, http://www.gsea-msigdb.org/gsea/msigdb, accessed on 17 June 2022) [38,39].

Proteomic profiles of ICR-SS-1 and the other two synovial sarcoma cell lines were compared by a two-tailed *t*-test in Perseus. For this analysis, the log2 transformed, quantile normalised dataset was used. Technical replicates for individual cell lines were kept as separate samples and proteins with no valid values across the three cell lines were removed. A permutation-based FDR threshold of 0.05 and artificial within-group variance of 0.1 were applied in Perseus to identify significant differentially regulated proteins [36]. Lists of identified up- and down-regulated proteins were further analysed by g:Profiler as described above.

### 2.9. Drug Screen and Dose Response Assays

ICR-SS-1 (2000/well), HS-SY-II (3000/well), and SYO-1 (2000/well) cells were seeded in clear 96-well, flat-bottom plates (Corning Inc., Corning, NY, USA). Plates were incubated for 24 h before replacing media with a panel of small molecule inhibitors at a concentration of 500 nM for all drugs except NVP-AUY922, which was at a concentration of 50 nM (details and source of inhibitors are shown in Supplementary Table S1). After 72 h, cell viability was determined using CellTitre-Glo (Promega, Madison, WI, USA), following the manufacturer’s instructions. Dose response assays were conducted by seeding ICR-SS-1 (2000/well), HS-SY-II (3000/well), and SYO-1 (2000/well) cells in clear 96-well, flat-bottom plates (Corning). Plates were incubated for 24 h, after which the medium was replaced with increasing concentrations of doxorubicin hydrochloride (Sigma Aldrich) or pazopanib (LC Laboratories) at the indicated dose. Data points from dose response assays were used to fit a four-point non-linear regression curve via Graphpad Prism and the drug screen data were subjected to hierarchical clustering using Perseus software [35] with Euclidean distance as the distance metric.

## 3. Results

### 3.1. Authentication of Established Cell Line

We established a cell line ICR-SS-1 which was derived from a PDX model of synovial sarcoma J000104314 deposited in The Jackson Laboratory biorepository. The ICR-SS-1 cell line was authenticated by short tandem repeat (STR) analysis at 16 loci (Table 6). There was a 100% match between the cell line and originating J000104314 PDX tumour, confirming that the cell line was established from the original tumour and that there was no contamination with other cell lines.

### 3.2. Molecular Assessment of SS18::SSX Fusion Status

**Figure 2:**
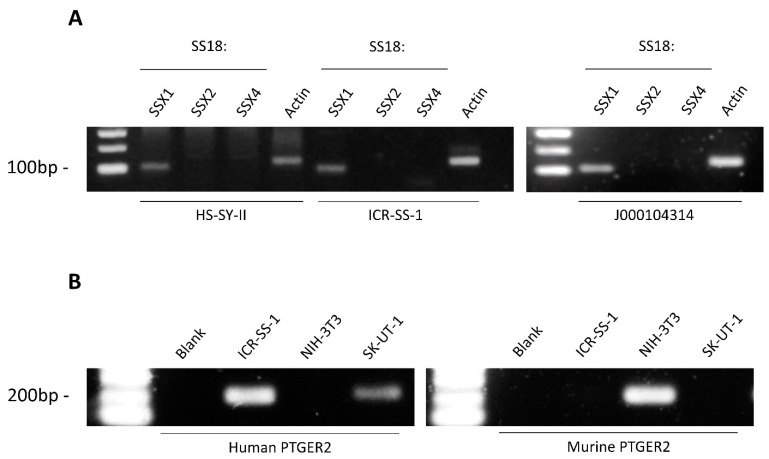
(**A**) PCR analysis of *SS18::SSX1*, *SSX2*, or *SSX4* gene fusions in ICR-SS-1 cell line and J000104314 PDX tissue. The HS-SY-II cell line is used as a positive control. (**B**) PCR analysis of human and murine *PTGER2* in ICR-SS-1 cell line. The NIH-3T3 cell line is used as a murine positive control and SK-UT-1 cell line as a human positive control.

Synovial sarcoma is characterised by the *SS18::SSX* fusion gene. To evaluate if this fusion was present and retained in the ICR-SS-1 cell line, we undertook PCR analysis of *SS18* and the three different possible fusion partners (*SSX1*, *SSX2*, *SSX4*). Using the HS-SY-II cell line, which has the *SS18::SSX1* fusion gene as a positive control [40], we show that both ICR-SS-1 and its originating PDX tumour J000104314 harbour the *SS18::SSX1* fusion gene (Figure 2A), confirming the diagnosis of synovial sarcoma.

### 3.3. Verification That ICR-SS-1 Is Not Contaminated with Murine Cells

Given that the ICR-SS-1 cell line was derived from a PDX model, we sought to verify that the cell line was not contaminated with murine cells originating from the host. We performed PCR analysis with species-specific primers for the prostaglandin E receptor 2 (*PTGER2*) gene as previously described [41]. Using the SK-UT-1 leiomyosarcoma cells as a human cell line control and NIH-3T3 cells as a murine cell line control, we show that ICR-SS-1 only expressed the human and not the murine version of *PTGER2* (Figure 2B), confirming that this cell line is comprised of a pure population of human cells.

### 3.4. In Vitro Characteristics of the Cell Line

**Figure 3:**
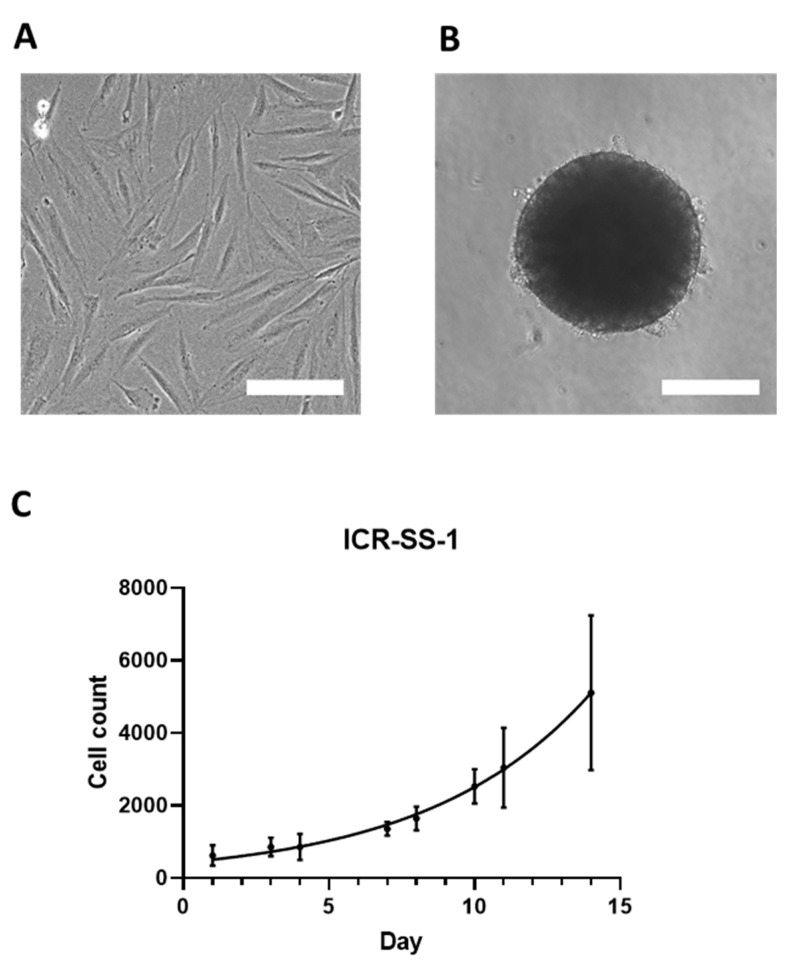
(**A**) Phase-contrast image of ICR-SS-1 cells in 2D. Scale bar = 200 µm. (**B**) Brightfield image of ICR-SS-1 spheroid. Scale bar = 200 µm. (**C**) Cell count of ICR-SS-1 cell line over 14 days. *n* = 3.

The ICR-SS-1 cell line is adherent and composed of elongated spindle cells when grown in 2D (Figure 3A). The cells have been grown for 18 passages over 4 months. Furthermore, the cells are able to form 3D spheroids when grown in ultra-low attachment plates (Figure 3B). Assessment of the growth rate of ICR-SS-1 showed exponential growth with a population doubling time of 93 h (Figure 3C).

### 3.5. Comparative Proteomic Profiling by Mass Spectrometry

**Figure 4:**
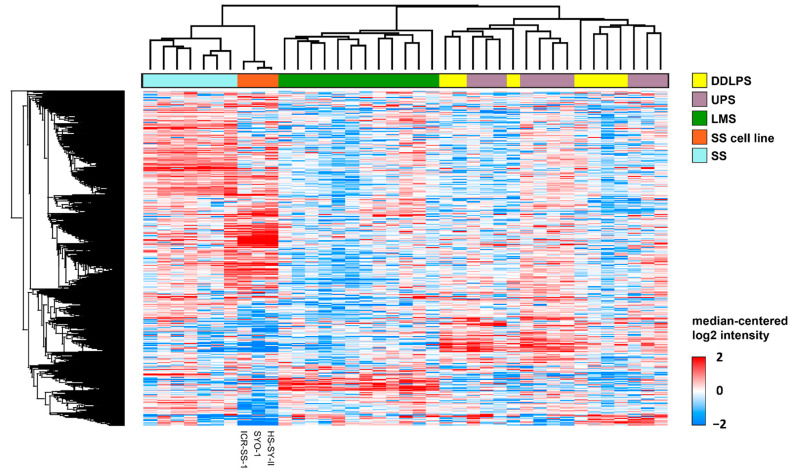
Heatmap displaying hierarchical clustering of proteomic data from synovial sarcoma cell lines and patient specimens of four histological subtypes (dedifferentiated liposarcoma—DDLPS, undifferentiated pleomorphic sarcoma—UPS, leiomyosarcoma—LMS, and synovial sarcoma—SS). Proteomic data were clustered with two-way unsupervised clustering based on Pearson’s correlation coefficient.

We have previously undertaken proteomic profiling by SWATH-mass spectrometry of tumour specimens from a cohort of soft tissue sarcoma patients (*n* = 36) comprising four histological subtypes including synovial sarcoma [31]. This study showed that different sarcoma histological subtypes are characterised by distinct proteomic profiles [42,43]. To determine if ICR-SS-1 faithfully recapitulates the molecular characteristics found in human synovial sarcoma tumours, we subjected the ICR-SS-1, as well as two established commercial synovial sarcoma cell lines (SYO-1 and HS-SY-II), to proteomic analysis by SWATH-mass spectrometry. Integrating these cell line proteomic data with the cohort of 36 patients across 4336 proteins shows that the three synovial sarcoma cell lines cluster closely together with the synovial sarcoma patient specimens, but separate from the three other histological subtypes (leiomyosarcoma, dedifferentiated liposarcoma, and undifferentiated pleomorphic sarcoma) (Figure 4). These data provide evidence that ICR-SS-1 faithfully reproduces the key molecular features found in synovial sarcoma patient specimens which are distinct from other subtypes.

**Figure 5:**
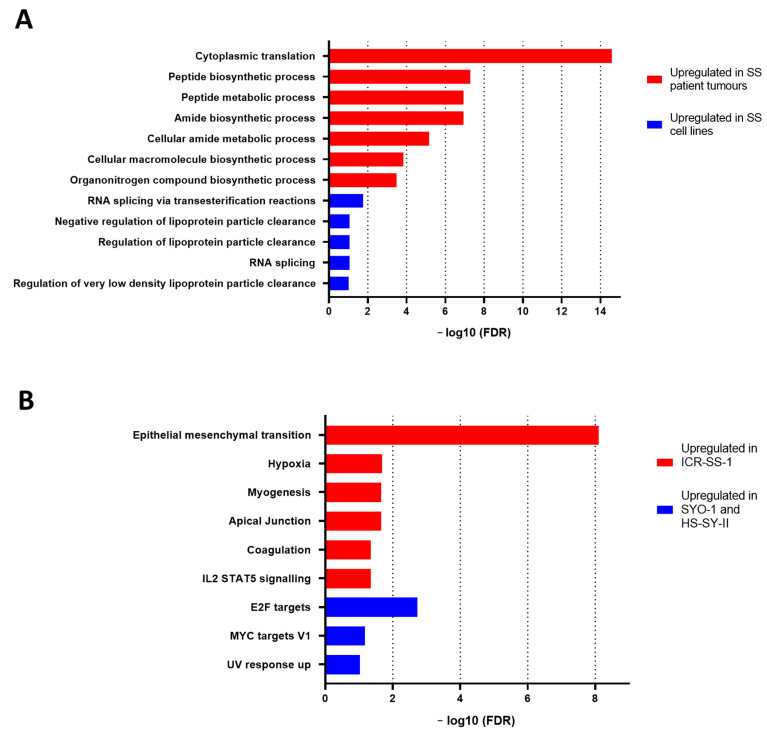
(**A**) Over-representation analysis plot showing biological processes that are upregulated in synovial sarcoma (SS) patient tumours samples (red) and in cell lines (blue) after mutual comparison. (**B**) Over-representation analysis plot showing hallmark pathways upregulated in ICR-SS-1 (red) and in SYO-1 with HS-SY-II (blue) after mutual comparison. FDR is false discovery rate.

Although the cell lines clustered together with the synovial sarcoma patient specimens, there were differences between the proteomic profiles of the human tumours and cell lines (Figure 4). We undertook a significance analysis of microarray (SAM) to identify the proteins that are significantly different between the synovial sarcoma cell lines and tumour specimens. Over-representation analysis using the g:Profiler tool showed that biological processes involved in protein translation and biosynthesis were significantly enriched in patient specimens compared to the cell lines (Figure 5A). These ontologies include the “cytoplasmic translation”, “peptide biosynthetic”, “peptide metabolic”, “amide biosynthesis”, and “organonitrogen compound biosynthesis” ontologies. In contrast, ontologies upregulated in cell lines versus patient tissue specimens comprised proteins involved in the regulation of lipoprotein particle clearance and RNA splicing.

We further sought to better understand the biological differences between ICR-SS-1 and the two commercially available synovial sarcoma cell lines SYO-1 and HS-SY-11 by performing a *t*-test to identify the proteins that are significantly different between these two groups. g:Profiler over-representation analysis finds that ICR-SS-1 cells had an upregulation of proteins involved in epithelial-to-mesenchymal transition (EMT), while the commercial SS cell lines showed elevated levels of proteins which are E2F targets (Figure 5B). These data demonstrate that there are distinct biological pathways operating in ICR-SS-1 versus the SYO-1 and HS-SY-II cell lines.

### 3.6. Characterisation of Response to Anticancer Agents

**Figure 6:**
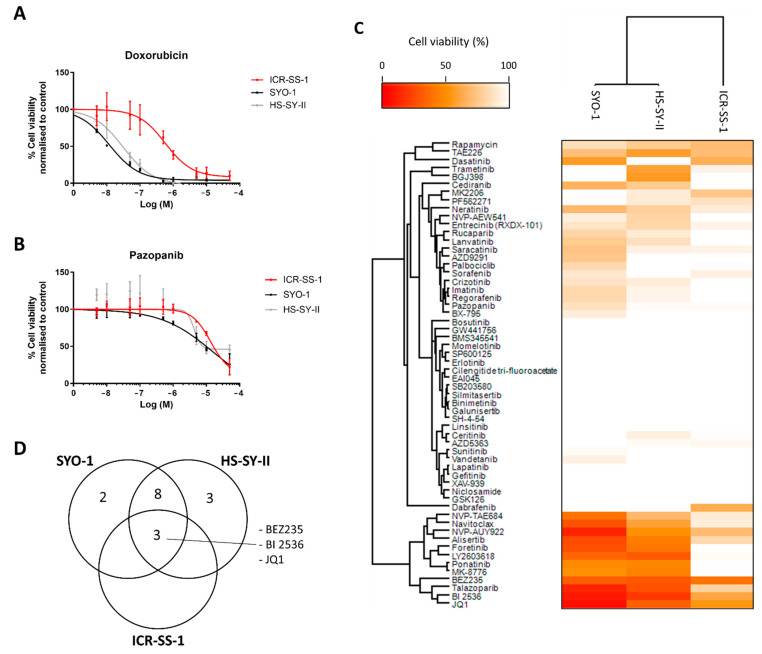
(**A**) Dose–response curves of ICR-SS-1, SYO-1, and HS-SY-II treated with doxorubicin. *n* = 3. (**B**) Dose–response curves of ICR-SS-1, SYO-1, and HS-SY-II treated with pazopanib. *n* = 3. (**C**) A heatmap of drug response of ICR-SS-1, SYO-1, and HS-SY-II cell lines upon treatment with 58 targeted small molecule inhibitors at 500 nM or in the case of NVP-AUY922, 50 nM. *n* = 3. (**D**) Venn diagram of shared and unique targeted inhibitor sensitivities (<65% viability) across the three cell lines.

The current standard of care for synovial sarcoma in the first line is anthracycline therapy. We therefore assessed the effect of doxorubicin on ICR-SS-1 compared to SYO-1 and HS-SY-II. ICR-SS-1 was significantly more resistant to doxorubicin (ICR-SS-1 IC50 = 613 ± 299 nM) compared to the other cell lines (SYO-1 IC50 = 13 ± 1 nM, HS-SY-II IC50 = 31 ± 14 nM) (*p* < 0.01) (Figure 6A). Pazopanib is a tyrosine kinase inhibitor which is approved for the treatment of SS following failure of doxorubicin. Dose–response assessment finds that all three cell lines are resistant to this drug (IC50 > 5 µM) (Figure 6B). Furthermore, we undertook a comparative screen of the three cell lines to a panel of 58 small molecule inhibitors that target a broad range of oncogenic signalling pathways (Supplementary Table S1 and Figure 6C,D). The screen showed that ICR-SS-1 had a distinct drug response profile compared to the other two synovial sarcoma cell lines and was generally more resistant to the vast majority of small molecule inhibitors tested (Figure 6C). An evaluation of shared vulnerabilities across all three synovial cell lines identified three compounds, the dual PI3K-mTOR inhibitor NVP-BEZ235, the PLK1 inhibitor BI 2536, and the BET bromodomain inhibitor JQ1 (Figure 6D), suggesting that targeting these pathways may have broad therapeutic utility in synovial sarcomas.

## 4. Discussion

Synovial sarcoma is a rare cancer type with poor outcomes in the advanced setting despite multidisciplinary clinical management. There is a lack of effective systemic therapies for these patients and therefore an urgent need to develop new approaches to tackle this disease. Key to the identification of novel agents is the availability of well-characterised preclinical cell line models. To date, only five synovial sarcoma cell lines are available in public biorepositories [14], all of which have been cultured for decades. Here, we present and characterise a new synovial sarcoma cell line ICR-SS-1, which has been established from a PDX model held in The Jackson Laboratory biorepository. This cell line retains the *SS18::SSX1* fusion gene and faithfully recapitulates the molecular features of human synovial sarcoma tumours as shown by mass spectrometry analysis.

Despite being driven by a single translocation (the *SS18::SSX* fusion gene), it is well-established that in synovial sarcoma patients there is a wide heterogeneity in observed clinical responses to systemic therapies. For instance, only a subset of advanced synovial sarcoma patients benefits from treatment with chemotherapeutic agents such as doxorubicin and trabectedin [44,45,46]. The mechanistic basis for this heterogeneity is currently unknown and there are no predictive biomarkers available for stratification in order to rationally target the right drugs to the appropriate patient population. Notably, our data show that the ICR-SS-1 cell line is significantly more resistant to doxorubicin compared to two established synovial cell lines SYO-1 and HS-SY-II. Our proteomic analysis finds that when compared to the established cell lines, the ICR-SS-1 line shows significantly upregulated expression of proteins involved in EMT. In line with this observation, previous studies have shown that the induction of EMT drives doxorubicin resistance in multiple cancer types [47]. This suggests that ICR-SS-1 could serve as a useful model to study the biology of anthracycline-resistant synovial sarcoma and identify new salvage therapies, such as those that target the EMT pathway, following the failure of doxorubicin treatment.

By subjecting ICR-SS-1 to a targeted small molecule inhibitor panel and comparing the drug responses to the two other synovial sarcoma cell lines, we identified agents from three different drug target classes which are effective in all three cell lines. Of these, both SYO-1 and HS-SY-II have previously been shown to be sensitive to PLK-1 inhibition [48] while BET bromodomain inhibitors [49]. In addition, we show that the dual PI3K-mTOR inhibitor NVP-BEZ235 (also known as dactolisib) reduces the cell viability of ICR-SS-1 and the two other synovial sarcoma cell lines. Our data are consistent with previous reports of short-term patient-derived sarcoma modelling studies which demonstrate that tumour cells from synovial sarcoma patients are sensitive to drugs that block this pathway [50,51]. Taken together, our data suggest that targeting the PI3K-mTOR pathway may have utility particularly in the context of chemotherapy-resistant synovial sarcoma.

## 5. Conclusions

In summary, we have developed and characterised a matched PDX-derived cell line for synovial sarcoma which will add to the arsenal of preclinical tools available for the study of this rare cancer. In particular, the ICR-SS-1 cell line model may be valuable for studies focused on the biology of anthracycline-resistant sarcoma and identifying novel ways to tackle this clinically challenging problem.

## Figures and Tables

**Table 1:** Primer sequences for *SS18::SSX* and *ACTB*.

PCR Amplicon	Forward Primer	Reverse Primer
*SS18::SSX1*	5′-AGACCAACACAGCCTGGACCAC-3′	5′-ACACTCCCTTCGAATCATTTTCG-3′
*SS18::SSX2*	5′-AGACCAACACAGCCTGGACCAC-3′	5′-GCACTTCCTCCGAATCATTTC-3′
*SS18::SSX4*	5′-AGACCAACACAGCCTGGACCAC-3′	5′-GCACTTCCTTCAAACCATTTTCT-3′
*ATCB*	5′-GACAGGATGCAGAAGGAGATCAC-3′	5′-TGATCCACATCTGCTGGAAGGT-3′

**Table 2:** Thermocycler conditions for *SS18::SSX*.

*SS18::SSX*			
PCR Step	Time	Temperature	Comments
Denaturation	7 min	95 °C	
Touchdown Amplification	45 s	94 °C	10 cycles, reducing annealing temperature by 1 each cycle, from 66 to 57
	45 s	66 °C	
	1 min 30 s	72 °C	
Amplification	45 s	94 °C	30 cycles
	45 s	56 °C	
	1 min 30 s	72 °C	
Final Extension	5 min	72 °C	

**Table 3:** Thermocycler conditions for *ATCB*.

*ATCB*			
PCR Step	Time	Temperature	Comments
Denaturation	2 min	95 °C	
Amplification	15 s	95 °C	40 cycles
	15 s	60 °C	
	1 min	72 °C	
Final Extension	5 min	72 °C	

**Table 4:** Primer sequences for *PTGER2*.

PCR Amplicon	Forward Primer	Reverse Primer
Human *PTGER2*	5′-GCTGCTTCTCATTGTCTCGG-3′	5′-GCCAGGAGAATGAGGTGGTC-3′
Mouse *PTGER2*	5′-CCTGCTGCTTATCGTGGCTG-3′	5′-GCCAGGAGAATGAGGTGGTC-3′

**Table 5:** Thermocycler conditions used for human or mouse *PTGER2* amplification.

*SS18::SSX*			
PCR Step	Time	Temperature	Comments
Denaturation	5 min	98 °C	
Amplification	5 s	98 °C	40 cycles
	5 s	60 °C	
	20 s	72 °C	
Final Extension	1 min	72 °C	

**Table 6:** Short tandem repeat profile of J000104314 PDX tumour and ICR-SS-1 cell line.

	Samples	
Locus	J000104314	ICR-SS-1
D8S1179	13, 13	13, 13
D21S11	29, 31.2	29, 31.2
D7S820	7, 8	7, 8
CSF1PO	10, 12	10, 12
D3S1358	17, 17	17, 17
TH01	6, 9.3	6, 9.3
D13S317	12, 14	12, 14
D16S539	9, 12	9, 12
D2S1338	20, 23	20, 23
D19S433	13, 15	13, 15
vWA	16, 16	16, 16
TPOX	8, 9	8, 9
D18S51	12, 15	12, 15
AMEL	X, Y	X, Y
D5S818	11, 13	11, 13
FGA	24, 24	24, 24

## Supplementary Materials

The following supporting information can be downloaded at: https://www.mdpi.com/article/10.3390/cells11152418/s1, Table S1: Small molecule inhibitor screen with associated primary target(s) and supplier.
