# Supplementary material for: Characterisation of a Novel Cell Line (ICR-SS-1) Established from a Patient-Derived Xenograft of Synovial Sarcoma
Source: Cells. 2022 Aug 4;11(15):2418. doi: 10.3390/cells11152418 (PMC9368503; doi:10.3390/cells11152418)
Supplement: Supplementary file 1 [file cells-11-02418-s001.zip › cells-1704223-supplementary.pdf]

Table S1. Small molecule inhibitor screen with associated primary target(s) and supplier.

Inhibitor	Primary Target(s)	Supplier
**Lenvatinib**	Broad Spectrum: RTKs	LC Labs
**Sunitinib**	Broad Spectrum: RTKs	LC Labs
**Pazopanib**	Broad Spectrum; RTKs	LC Labs
**Ponatinib**	Broad spectrum; RTKs	LC Labs
**Regorafenib**	Broad Spectrum: RTKs	LC Labs
**Bosutinib**	Src, Abl	LC Labs
**Dasatinib**	Abl, Src, c-Kit	LC Labs
**Saracatinib**	Src	Selleck Chemicals
**Ceritinib**	ALK	LC Labs
**Crizotinib**	c-Met, ALK	LC Labs
**NVP-TAE684**	ALK	Selleck Chemicals
**AZD9291**	EGFR	Selleck Chemicals
**EAI045**	EGFR	Selleck Chemicals
**Erlotinib**	EGFR	LC Labs
**Gefitinib**	EGFR	LC Labs
**Lapatinib**	EGFR, ErbB2	Selleck Chemicals
**Neratinib**	HER2, EGFR	LC Labs
**BGJ398**	FGFR1/2/3	Selleck Chemicals
**Linsitinib**	IGF-1R	LC Labs
**NVP-AEW541**	IGF-1R, InsR	Selleck Chemicals
**Imatinib**	v-Abl, c-Kit, PDGFRα	LC Labs
**Cediranib**	VEGFR	LC Labs
**Foretinib**	HGFR, VEGFR	LC Labs
**Sorafenib**	Raf-1, B-Raf, VEGFR2	LC Labs
**Vandetanib**	VEGFR2	LC Labs
**BI2536**	Plk1	Selleck Chemicals
**BX-795**	PDK1	Sigma-Aldrich
**NVP-BEZ235**	PI3K, mTOR	LC Labs
**Rapamycin**	mTOR	LC Labs
**Binimetinib**	MEK1/2	LC Labs
**Trametinib**	MEK1/2	LC Labs
**Dabrafenib**	BRAFV600	Selleck Chemicals
**SB203580**	p38 MAPK	LC Labs
**SP600125**	JNK	LC Labs
**NVP-AUY922**	HSP90	LC Labs
**AZD5363**	Akt1/2/3	Selleck Chemicals
**MK2206**	Akt1/2/3	Selleck Chemicals
**Momelotinib**	JAK1/2	Selleck Chemicals
**Niclosamide**	STAT3	Selleck Chemicals
**SH-4-54**	STAT	Selleck Chemicals
**Cilengitide Trifluoroacetate**	Integrin	Selleck Chemicals
**Navitoclax**	Bcl-xL, Bcl-2, Bcl-w	Selleck Chemicals
**Galunisertib**	TGFβ-R1	Selleck Chemicals
**Entrectinib**	TrkA/B/C, ROS1, ALK	Selleck Chemicals
**GW441756**	TrkA	Selleck Chemicals
**BMS345541**	IKK-1/2	Selleck Chemicals
**GSK126**	EZH2 Methyltransferase	Selleck Chemicals
**XAV-939**	Tankyrase-1/2	Selleck Chemicals
**PF562271**	FAK	Selleck Chemicals
**TAE226**	FAK	Selleck Chemicals
**Alisertib**	Aurora A	Selleck Chemicals
**JQ1**	BET Bromodomain	Selleck Chemicals

**LY2603618**	Chk1	Selleck Chemicals
**MK-8776**	Chk1	Selleck Chemicals
**Palbociclib**	CDK4/6	LC Labs
**Silmitasertib**	CK2	Selleck Chemicals
**Rucaparib**	PARP	LC Labs
**Talazoparib**	PARP	Selleck Chemicals
